# Effects of *Lactobacillus fermentum* CQPC04 on Lipid Reduction in C57BL/6J Mice

**DOI:** 10.3389/fmicb.2020.573586

**Published:** 2020-09-03

**Authors:** Ruokun Yi, Fang Tan, Xianrong Zhou, Jianfei Mu, Lin Li, Xiping Du, Zhennai Yang, Xin Zhao

**Affiliations:** ^1^Beijing Advanced Innovation Center for Food Nutrition and Human Health, Beijing Technology & Business University (BTBU), Beijing, China; ^2^Chongqing Collaborative Innovation Center for Functional Food, Chongqing Engineering Research Center of Functional Food, Chongqing Engineering Laboratory for Research and Development of Functional Food, Chongqing University of Education, Chongqing, China; ^3^Department of Public Health, Our Lady of Fatima University, Valenzuela, Philippines

**Keywords:** *Lactobacillus fermentum*, lipid reduction, C57BL/6J mice, oxidation, expression

## Abstract

Probiotics are functional foods that can effectively regulate lipid reduction and maintain body health. In this study, a strain of *Lactobacillus fermentum* CQPC04 (LF-CQPC04) isolated from traditional naturally fermented vegetables (Sichuan pickles) was studied, and its effects on lipid reduction in mice, as well as its mechanism of action, were observed. The results of this experiment show that LF-CQPC04 can reduce the abnormal weight gain and abnormal visceral index of mice caused by a high-fat diet. LF-CQPC04 can decrease TG (triglycerides), TC (total cholesterol), LDL-c (low-density lipoprotein cholesterol), AST (aspartate transaminase), ALT (alanine aminotransferase), and AKP (alkaline phosphatase) levels and increase HDL-c (high-density lipoprotein cholesterol) levels in the serum of high-fat mice. LF-CQPC04 can also decrease the levels of inflammatory cytokines, such as IL-6 (interleukin-6), IL-1β (interleukin-1 beta), TNF-α (tumor necrosis factor alpha), and IFN-γ (interferon gamma), and increase IL-4 and IL-10 levels in the serum of high-fat mice. The results of RT-qPCR (real-time quantitative polymerase chain reaction) and western blot experiments show that LF-CQPC04 can also down-regulate the expression of PPAR-γ (peroxisome proliferator-activated receptor gamma), C/EBP-α (CCAAT/enhances binding protein alpha) mRNA, and protein in the liver tissue of high-fat mice, while up-regulating the expression of Cu/Zn-SOD (copper/zinc superoxide dismutase), Mn-SOD (manganese superoxide dismutase), CAT (catalase), CYP7A1 (cholesterol 7 alpha hydroxylase), PPAR-α (peroxisome proliferator-activated receptor alpha), CPT1 (carnitine palmitoyl transferase 1), LPL (lipoprotein lipase), and ABCA1 (ATP-binding cassette transporter A1). Moreover, LF-CQPC04 shows stronger effects in regulating lipid reduction in mice than L-carnitine and commercial LB (*Lactobacillus delbrueckii* subsp. *Bulgaricus*) bacteria. LF-CQPC04 is beneficial for lipid reduction in animals and has good probiotic potential.

## Introduction

Obesity is a state in which body fat, especially triglyceride (TG; triacylglycerol), accumulates excessively. The common cause of obesity is long-term excessive energy intake that exceeds the body’s energy consumption. Excess energy in the body is converted into fat, and excessive fat accumulation leads to nutritional metabolic disorders (Lee et al., 2018). In the 1990s, the World Health Organization declared obesity as a chronic metabolic disease caused by multiple factors (Cai, 2013). However, only in recent years have people gradually realized that it is a serious social health problem. According to predictions from the International Diabetes Federation, by 2035, the global number of diabetic patients will increase to 590 million (Tong and Liu, 2017). Obesity is associated with hypertension, coronary heart disease, stroke, hyperlipidemia, sleep apnea syndrome, type 2 diabetes, gout, bone and joint diseases, decreased reproductive function, and certain cancers (Baretiæ, 2012). The survey found that the prevalence of obese and overweight people has risen rapidly in recent years (Tong and Liu, 2017). Thus, it is important to raise awareness regarding obesity and promote weight loss going forward. Many obese individuals lose weight through dieting or taking drugs. Others may achieve a significant weight loss with drugs, which may lead to a sharp decrease in their immunity. Many achieve weight loss using unscientific approaches. Such unscientific means might reduce muscle mass rather than fat. This is the common weight loss method of the unadvised public (Stern, 1984). Moreover, drug treatments, surgical treatments, and lifestyle interventions are common approaches used to treat obesity. The former is the simplest and safest method. However, since the effects of drugs require time and consistent use from patients, there are very few successful cases of weight loss using this method (Wilding, 1997). There are certain side effects in almost all of the weight loss drugs available on the market. Moreover, individuals might experience a rebound phenomenon after drug withdrawal. Due to factors, including drug safety, their application is restricted (Kakkar and Dahiya, 2015).

Probiotics are a type of microorganism with clear biological activity. They are beneficial to the human body. When ingested, they promote the absorption of nutrients and maintain intestinal health by regulating the immune function of the host’s mucosa, as well as balancing the intestinal flora (Guarner and Schaafsma, 1998). Some lactic acid bacteria can play special physiological functions as probiotics. They can promote growth, regulate normal flora in the gastrointestinal tract, and maintain micro-ecological balance, thereby improving gastrointestinal functions, food digestibility, biological potency, and body immunity. Lactic acid bacteria with probiotic functions can also reduce serum cholesterol, control endotoxins, and inhibit the growth of spoilage bacteria in the intestinal tract (Brooks and Kalmokoff, 2012; Boumis et al., 2018; Peters et al., 2019; Santacroce et al., 2019). Researchers have discovered hundreds of microorganisms in the intestines of healthy people. These microorganisms are involved in digestion and energy production. The energy intake of the human body may be affected by microorganisms in the intestinal tract. Some intestinal microbes provide energy more efficiently than others, leading to weight gain. Some individuals absorb nutrients more effectively than others (Fooks et al., 1999). Some live lactic acid bacteria, which are implanted in the intestine, multiply to maintain a specific volume. They participate in long-term sugar-lipid metabolism and cholesterol-lipid metabolism, block the formation of fat, and accelerate the oxidative decomposition of accumulated fat in the body, generating long-term weight loss without producing any side effects (Tennyson and Friedman, 2008).

A recent study has shown that intestinal microbes are closely related to obesity, and they also play an important role in human metabolism (Turnbaugh et al., 2006). Intestinal microorganisms are closely related to human energy metabolism and balance. Oxidative stress caused by a high-fat diet can lead to an imbalance of intestinal microorganisms, followed by a change in the composition of intestinal microorganisms (Hildebrandt et al., 2009). Intestinal microbiota can affect the energy intake of the host via diet and energy storage (Jumpertz et al., 2011). Metabolites of intestinal microbiota, such as short chain fatty acids and lipopolysaccharides, also play a very important role in the process of fat accumulation by affecting lipid metabolism, energy metabolism, inflammation, and appetite (Samuel et al., 2008; Sanz et al., 2008; Baothman et al., 2016). Therefore, the change in intestinal flora can be used as a weight gain prevention method, as well as an intervention method to reduce the amount of fat.

In China, the Sichuan pickle is a traditional fermented vegetable. Lactic acid bacteria is the most important fermenting microorganism it contains (Zhang et al., 2019). Lactic acid bacteria isolated from Sichuan pickles were proven to have intestinal protection effects, as well as good prevention and intervention effects on constipation and colitis (Liu et al., 2019). In this study, a strain of *Lactobacillus fermentum* CQPC04 (LF-CQPC04), isolated and identified by our research group, was selected to conduct experiments on its ability to reduce lipids in animals. Additionally, its mechanism of action was analyzed with molecular biology techniques.

## Materials and Methods

### Activation of Bacteria

LF-CQPC04 (patent deposit No. CGMCC 14493) was separated by our team from homemade naturally fermented pickles sold at a market in Nanan District, Chongqing, China. It was preserved after identification by 16S rRNA. LB was purchased from the China Center for Type Culture Collection (Wuhan, Hubei, China). At the beginning of the experiment, the strains were removed from glycerin cryopreservation tubes, and 1% (50 μL) of the strains were inoculated in 5 mL of MRS liquid medium. The strains were incubated for 16–24 h at 37°C in a water bath shaker. Fifty microliters of bacteria-containing medium was transferred to a clean tube and combined with 5 mL of MRS medium. The mixture was incubated for 16–24 h at 37°C in a water bath shaker. In order to preserve the strains, 0.5 mL of twice-activated bacteria-containing medium and 0.5 mL of 40% sterile glycerol were mixed well in a 2-mL centrifuge tube. The tube was sealed with a sealing film, labeled, and put aside for later use (Yi et al., 2019).

### Gram Staining of *Lactobacillus fermentum*

A total of 1.5 mL of bacteria-containing culture medium activated twice in a 2-mL centrifuge tube and centrifuged at 3000 r/min for 10 min was taken. To the centrifuge tube, 1.5 mL of physiological saline was added to make a bacterial suspension. A drop of bacterial suspension was dropped on a glass slide, and it was spread out into a circle with a diameter of 1 cm. The circle was dried on an alcohol lamp. A drop of CrFStal violet staining solution was added, ensuring that it covered the circle containing the bacterial suspension. After 1 min, the glass slide was gently rinsed with purified water and dried naturally. Next, the circle was covered with iodine solution for 1 min. It was subsequently washed with water and dried with absorbent paper. Three drops of decolorizing solution were applied, and the bacterial sample was decolorized for 30−60 s. The circle was washed with water, dried, stained with safranine staining solution for 30 s, rinsed with purified water, and dried with absorbent paper. The sample was completely dried prior to microscopic examination (Yi et al., 2019).

### Tolerance of Lactic Acid Bacteria to 0.3% Bile Salt

Pig bile salt was added to MRS-THIO medium (MRS broth containing 0.2% sodium thioglycolate) to make it 0.3% and sterilized at 121°C for 15 min. The activated 5 mL strain was inoculated into MRS-THIO medium without bile salt (0.0%) and MRS-THIO medium containing 0.3% bile salt with 2% (v/v) inoculum volume. Blank medium (MRS-THIO medium without bacteria) was used as the control, and the OD_600_ nm values of the above media with different concentrations were determined after 24 h incubation at 37°C. The tolerance of the strain to bile salt was calculated according to the formula: bile salt tolerance (%) = (OD_600_ of 0.3% bile salt medium − OD_600_ of blank medium)/(OD_600_ of 0.0% bile salt medium − OD_600_ of blank medium) × 100.

### Artificial Tolerance Test of Gastric Juice

The artificial gastric juice was composed of 0.2% NaCl and 0.35% pepsin. According to the corresponding mass volume ratio, the NaCl and pepsin required for the test were weighed and prepared. The pH value of the prepared artificial gastric juice was adjusted to 3.0 with 1 mol/L HCl. The artificial gastric juice was filtered and sterilized by a 0.22-μM filter membrane. On an ultra-clean table, 5 mL of cultured bacteria were transferred to 10-mL sterile centrifuge tubes, and centrifuged at 3000 r/min for 10 min. The upper culture medium was discarded, and the bacteria were collected. An equal volume (5 mL) of sterile saline was added to make a bacterial suspension. Then, 1 mL of bacterial suspension was mixed with 9 mL of artificial gastric juice at pH 3.0. At this time, 1 mL of the above mixture was taken as the sample of artificial gastric juice for 0 h, and the remaining 9 mL of the mixture was placed in a constant temperature water bath shaker (37°C, 150 r/min) for 3 h. The samples of 0 h and 3 h were diluted 10-fold, and a suitable gradient was selected. The number of viable bacteria was determined by plate coating method and culturing on MRS solid medium at 37°C for 48 h. The survival rate (%) was calculated according to the formula: survival (%) = 3 h viable count (CFU/mL)/0 h viable count (CFU/mL) × 100.

### Mice Grouping and Experiment

Sixty 6-week-old C57BL/6J mice (half male and half female, Chongqing Medical University, Chongqing, China) were randomly divided into six groups, with 10 mice in each group. As shown in Tables 1, 2, the mice were grouped and fed with experimental samples for 8 weeks. Their weight, food intake, and water intake were recorded daily. After 8 weeks, the mice were weighed after fasting for 12 h, and blood was taken from their eyeballs. The mice were sacrificed by cervical dislocation. Their livers and epididymal fat tissues were removed for later use.

**TABLE 1:** Grouping and treatment of experimental mice.

**Group**	**Feed**	**Treatment**
Normal	LFD	Untreated
Control	HFD	Untreated
L-carnitine	HFD	200 mg/kg L-carnitine
LF-CQPC04-L	HFD	1.0 × 10^8^ CFU/mL bacterial fluid
LF-CQPC04-H	HFD	1.0 × 10^9^ CFU/mL bacterial fluid
LB	HFD	1.0 × 10^9^ CFU/mL bacterial fluid

*LF-CQPC04, *Lactobacillus fermentum* CQPC04; LB, *Lactobacillus delbrueckii* subsp. *Bulgaricus*; LFD, low-fat diet; HFD, high-fat diet.*

**TABLE 2:** Composition formula of mouse feed.

**Diet ingredients (%)**	**LFD (%)**	**HFD (%)**
Corn flour	24.89	7.79
Bran	15.00	27.00
Wheatmeal	7.00	/
Soybean meal	18.50	22.00
Sucrose	16.38	20.00
Lard	1.02	19.50
Premix	17.21	23.71

*Premix: trace mineral, elements, vitamins, synthetic amino acids.*

### Determination of Organ Indicators

The mice were fasted for 12 h and their weight was recorded before sampling blood from the eyeballs. The mice were dissected, and their livers and epididymal fat were removed and weighed. The organ index was calculated according to the following formula: organ index (%) = organ weight (g)/mouse weight (g) × 100.

### Observation of the H&E Slices of Liver and Epididymis

H&E staining was used to make pathological slices of the liver and epididymal fat (Li et al., 2019). The pathological changes in the livers and epididymal fat were observed under an electron microscope (BX43; Olympus, Tokyo, Japan).

### Determination of TG, TC, HDL-C, LDL-C, AKP, AST, and ALT Levels in Serum and Liver Tissues

Whole blood collected from mice was added to an anticoagulation blood vessel, and the upper serum was obtained after centrifugal separation. The liver tissue was homogenized with a tissue homogenizer, and the tissue homogenate was obtained from the upper layer after centrifugation (4000 rpm/min for 10 min). Serum and tissue homogenate indicators were measured according to the kit instructions (Nanjing Jiancheng Bioengineering Institute, Nanjing, China) (Zhu et al., 2019).

### Determination of the Levels of Serum Cytokines TNF-α, INF-γ, IL-6, IL-1β, IL-4, and IL-10

The above method was used to make the serum, and the levels of cytokines in the serum were determined according to the kit instructions (Nanjing Jiancheng Bioengineering Institute).

### RT-qPCR

For sample preparation, 50−100 mg of liver, colonic tissues, and 0.5 g mouse feces were removed, and 1 mL of Trizol reagent (Invitrogen, Carlsbad, CA, United States) was added to the sample separately. The tissue or feces was homogenized, and 200 μL of chloroform was added to each sample. The samples were mixed well, placed in a refrigerator at 4°C for 5 min, and then centrifuged at 14000 r/min for 15 min at 4°C. The supernatant RNA (about 500 μL) was transferred to a new centrifuge tube, and a corresponding amount (1:1 RNA) of isopropanol was added to each sample, with mixing. The sample was placed in a refrigerator at 4°C for 15 min. The sample was centrifuged at 14000 r/min for 20 min, and the supernatant was discarded to obtain the yellow-white RNA. Oligo Primer dT (1 μL, Thermo Fisher Scientific, Waltham, MA, United States) reagent was mixed with sterilized ultrapure water (10 μL). One microliter of the extracted RNA was added, and it was placed in a 65°C water bath for 5 min. Then, 4 μL of 5 × Reaction Buffer, 1 μL of Ribdock RNase Inhibitor (20 U/μL), and 2 μL of 100 mM dNTP (Thermo Fisher Scientific, Waltham, MA, United States) were mixed and added to the previous reaction system. To the mixed reaction system, 1 μL of Revert Aid M-MuLV (Thermo Fisher Scientific, Waltham, MA, United States) was added. The sample was mixed well and centrifuged at 14000 r/min, 4°C for 10 min. The sample was incubated at 42°C for 60 min and then incubated at 70°C for 5 min. Finally, the reaction system was mixed, without the template, and it was added to a 8-tube strip, with 19 μL for each tube (10 μL master, 1 μL of upstream and downstream primers, 7 μL ddH_2_O, Table 3). Then, 1 μL of template was added and mixed well. The tube was covered and incubated after mixing and centrifuging. The relative expression of the target gene was calculated according to the following formula:

**TABLE 3:** Sequences of primers in this study.

**Gene name**	**Sequence**
*Cu/Zn-SOD*	Forward: 5′-AGGTCGGTGTGAACGGATTTG-3′
	Reverse: 5′-GGGGTCGTTGATGGCAACA-3′
*Mn-SOD*	Forward: 5′-CAGACCTGCCTTACGACTATGG-3′
	Reverse: 5′-CCTTCTCTTCCTCCCCTCTCTTC-3′
*CAT*	Forward: 5′-GGAGGCGGGAACCCAATAG-3′
	Reverse: 5′-CCACCATGTTTCTTAGAGTGAGG-3′
*PPAR-*α	Forward: 5′-CCTCAGGGTACCACTACGGAGT-3′
	Reverse: 5′-GCCGAATAGTTCGCCGAA-3′
*PPAR-*γ	Forward: 5′-AGGCCGAGAAGGAGAAGCTGTTG-3′
	Reverse: 5′-TGGCCACCTCTTTGCTGTGCTC-3′
*CYP7A1*	Forward: 5′-AGCAACTAAACAACCTGCCAGTACTA-3′
	Reverse: 5′-GTCCGGATATTCAAGGATGCA-3′
*CPT1*	Forward: 5′-AAAGATCAATCGGACCCTAGACA-3′
	Reverse: 5′-CAGCGAGTAGCGCATAGTCA-3′
*LPL*	Forward: 5′-AGGGCTCTGCCTGAGTTGTA-3′
	Reverse: 5′-AGAAATCTCGAAGGCCTGGT-3′
*C/EBP-*α	Forward: 5′-TGGACAAGAACAGCAACGAGTAC-3′
	Reverse: 5′-GCAGTTGCCCATGGCCTTGAC-3′
*ABCA1*	Forward: 5′-AAAACCGCAGACATCCTTCAG-3′
	Reverse: 5′-CATACCGAAACTCGTTCACCC-3′
*TNF-*α	Forward: 5′-CTGAACTTCGGGGTGATCGG-3′
	Reverse: 5′-GGCTTGTCACTCGAATTTTGAGA-3′
*ZO-1*	Forward: 5′-GCCGCTAAGAGCACAGCAA-3′
	Reverse: 5′-GCCCTCCTTTTAACACATCAGA-3′
*Firmicutes*	Forward: 5′-TGAAACTYAAAGGAATTGACG-3′
	Reverse: 5′-ACCATGCACCACCTGTC-3′
*Bacteroides*	Forward: 5′-CRAACAGAATTAGATACCCT-3′
	Reverse: 5′-GGTAAGGTTCCTCGCGTAT-3′
*Akkermansia*	Forward: 5′-GGAGATTACTGCCCTGGCTCCTA-3′
	Reverse: 5′-CACTCATCGTACTCCTGCTTGTTGCTG-3′
*GAPDH*	Forward: 5′-ACCCAGAAGACTGTGGATGG-3′
	Reverse: 5′-ACACATTGGGGGTAGGAACA-3′

2^–ΔΔCt =^ 2^–[(Ct^ ^target^ ^gene – Ct^ ^housekeeping^ ^gene) – (Ct^ ^target^ ^gene – Ct^ ^housekeeping^ ^gene)]^ (Liu et al., 2019).

### Western Blot

For Western blot analysis, 100 mg of liver tissue were homogenized with 1 mL of RIPA and 10 μL of PMSF (Thermo Fisher Scientific, Waltham, MA, United States) at 12000 r/min. The sample was lysed at 4°C for 5 min and centrifuged at 12000 r/min for 15 min at 4°C. The intermediate protein layer solution was removed for protein quantification using a BCA protein quantification kit (Thermo Fisher Scientific, Waltham, MA, United States). The samples of each group were diluted to 50 μg/mL. The diluted protein was mixed with Sample Buffer at 4:1, heated at 100°C for 5 min, and cooled in an ice bath for 5 min. Acrylamide, Resolving Buffer, Stacking Buffer, distilled water, 10% APS, and TEMED were mixed in proportion to make SDS − PAGE separation gel and concentrated gel (Thermo Fisher Scientific, Waltham, MA, United States), which were then poured into a running gel plate for later use. Prestained Protein Ladder and samples were pipetted into the sample wells of the running gel plate, and the protein-loaded SDS − PAGE gel was subjected to vertical gel electrophoresis for 50 min. The PVDF membrane was transferred after activation with methanol for 1 min. After the membrane was transferred, the PVDF membrane was sealed with 1 × TBST solution containing 5% skim milk for 1 h. The sealed PVDF membrane (Thermo Fisher Scientific, Waltham, MA, United States) was then washed with 1 × TBST. The primary antibody was incubated at 25°C for 2 h. After washing the PVDF membrane five times with 1 × TBST, the secondary antibody (Thermo Fisher Scientific, Waltham, MA, United States) was incubated at 25°C for 1 h. Finally, the PVDF membrane was sprayed with Supersignal West Pico PLUS and placed in the imaging system for observation (Tanon 5200, Shanghai Tanon Technology Co., Ltd., Shanghai, China) (Yi et al., 2019).

### Data Processing and Statistical Analysis

Values of more than three independent experiments were averaged as the final experiment data. Intra-group variance was calculated using one-way ANOVA with SPSS 17.0 statistical software. *P* < 0.05 was considered to be significantly different.

## Results

### LF-CQPC04 Microscopic Examination

**FIGURE 1:**
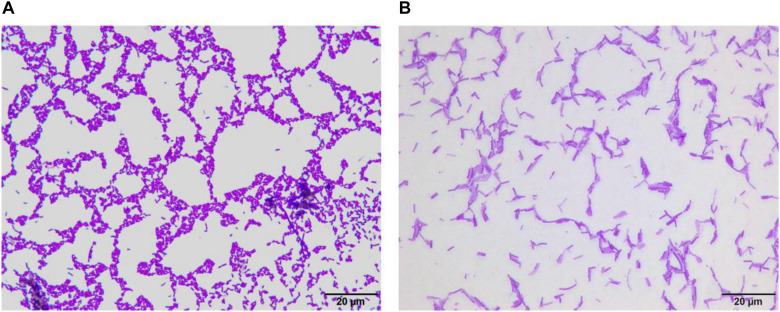
Morphology of *Lactobacillus fermentum* CQPC04 **(A)** and *Lactobacillus delbrueckii* subsp. *Bulgaricus* **(B)**.

After Gram staining of LF-CQPC04, the morphology of the lactic acid bacteria cells was observed under oil microscope. As shown in Figure 1, LF-CQPC04 cells were purple and Gram-positive. The cells were rod-shaped, and the morphology was normal. The control strain *Lactobacillus delbrueckii* subsp. *Bulgaricus* showed similar morphology. They were deemed usable for further animal experiments since no mixed bacteria were seen.

### *In vitro* Resistance Results of *Lactobacilli*

Our team sampled naturally fermented pickles from Sichuan and Chongqing regions of China, and 11 strains of lactic acid bacteria exhibited good resistance *in vitro* (Table 4). Seven strains of *Lactobacilli* were isolated at pH 3.0. The survival rate in artificial gastric juice exceeded 90%. The survival rate of two strains reached more than 80%, and the survival rate of the other two strains reached more than 60%. The survival rate of 11 strains of *Lactobacilli* in 0.3% bile salt was more than 10%, among which the survival rate of LF-CQPC04 was the highest, reaching 66.38%. This indicates a strong ability of the bacterium to tolerate bile salt. The remaining 10 strains of *Lactobacilli* grew well in 0.3% bile salt. Therefore, in this study, LF-CQPC04 was selected for further study in animal experiments.

**TABLE 4:** Resistance of lactic acid bacteria to artificial gastric juice and bile salt.

**Strains**	**Survival rate in pH 3.0 artificial gastric juice (%)**	**Survival in 0.3% bile salt (%)**
*Lactobacillus plantarum* CQPC01	90.43 ± 8.26	16.49 ± 0.39
*Lactobacillus plantarum* CQPC02	92.06 ± 6.91	17.3 ± 0.19
*Lactobacillus fermentum* CQPC03	104.12 ± 3.49	19.15 ± 5.05
*Lactobacillus fermentum* CQPC04	84.14 ± 6.06	66.38 ± 0.55
*Lactobacillus fermentum* CQPC05	84.19 ± 7.39	13.0 ± 0.58
*Lactobacillus fermentum* CQPC06	68.61 ± 2.20	23.49 ± 2.43
*Lactobacillus fermentum* CQPC07	91.76 ± 7.92	11.91 ± 0.20
*Lactobacillus fermentum* CQPC08	110.02 ± 3.53	12.39 ± 0.62
*Lactobacillus plantarum* CQPC09	68.49 ± 11.02	11.90 ± 2.37
*Lactobacillus plantarum* CQPC10	96.77 ± 2.83	11.23 ± 1.10
*Lactobacillus plantarum* CQPC11	107.69 ± 8.75	10.01 ± 2.17

### Status and Body Weights of the Mice

**FIGURE 2:**
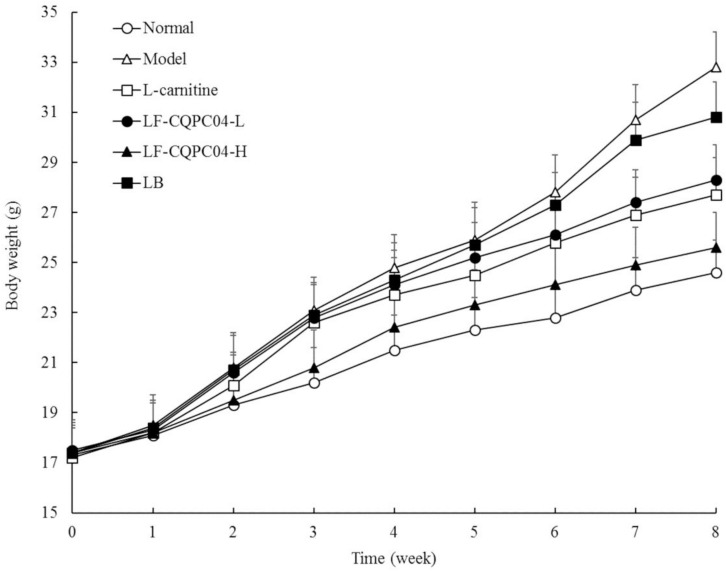
Body weight of mice. LF-CQPC04-L: mice treated with a low concentration of *Lactobacillus fermentum* CQPC04 (1.0 × 10^8^ CFU/kg); LF-CQPC04-H: mice treated with a high concentration of *Lactobacillus fermentum* CQPC04 (1.0 × 10^9^ CFU/kg); LB: Mice treated with *Lactobacillus delbrueckii* subsp. *Bulgaricus* (1.0 × 10^9^ CFU/kg).

After intragastric administration of LF-CQPC04 and LB samples, the daily condition of the mice was good. They showed quick response, had shiny and clean hair, and maintained a good mental state. Changes in the body weight of each group are shown in Figure 2. The body weight of the 8-week control group was the highest, and the normal group was the lowest. L-carnitine, LF-CQPC04-L, LF-CQPC04-H, and LB all inhibited weight gain in mice, to some extent. The body weight of the LF-CQPC04-H group was the closest to the normal group.

### Organ Index of Mice

The experimental results (Table 5) show that the liver index and epididymal index of the control group significantly increased, compared with the normal group. Compared with the control group, the liver index and epididymal index of L-carnitine group, high concentration group, low concentration group, and Bulgaria group all decreased significantly. The LF-CQPC04-H group shows the strongest decreasing trend.

**TABLE 5:** Organ indices of mice in each group.

**Group**	**Liver index**	**Epididymal adipose index**
Normal	2.12 ± 0.05^f^	1.20 ± 0.04^f^
Control	4.97 ± 0.15^a^	3.09 ± 0.11^a^
L-carnitine	3.51 ± 0.06^d^	2.02 ± 0.04^d^
LF-CQPC04-L	3.89 ± 0.06^c^	2.39 ± 0.06^c^
LF-CQPC04-H	2.89 ± 0.06^e^	1.69 ± 0.05^e^
LB	4.13 ± 0.08^b^	2.81 ± 0.06^b^

*Values presented are the mean ± standard deviation (*n* = 10/group). ^a–f^Mean values with different letters in the same column are significantly different (*P* < 0.05) according to Duncan’s multiple range test. LF-CQPC04-L: mice treated with a low concentration of *Lactobacillus fermentum* CQPC04 (1.0 × 10^8^ CFU/kg); LF-CQPC04-H: mice treated with a high concentration of *Lactobacillus fermentum* CQPC04 (1.0 × 10^9^ CFU/kg); LB: Mice treated with *Lactobacillus delbrueckii* subsp. *Bulgaricus* (1.0 × 10^9^ CFU/kg).*

### TG, TC, HDL-c, and LDL-c Levels in Serum and Liver Tissue of Mice

From Table 6, TG, TC, and LDL-c levels in the control group were significantly higher than those in the normal group (*P* < 0.05), and HDL-c level was significantly lower than that in the normal group (*P* < 0.05). TG, TC, and LDL-c levels of L-carnitine, LF-CQPC04-L, LF-CQPC04-H, and LB groups were all lower than the control group, while the HDL-c level was higher than the control group. Among them, TG, TC, and LDL-c levels of the LF-CQPC04-H group were only higher than that of the normal group, and its HDL-c level was only lower than that of the normal group.

**TABLE 6:** Serum and liver tissue levels of TC, TG, HDL-c and LDL-c in mice.

**Group (serum)**	**TC (mmol/L)**	**TG (mmol/L)**	**HDL-c (mmol/L)**	**LDL-c (mmol/L)**
Normal	1.82 ± 0.03^f^	0.11 ± 0.02^e^	1.55 ± 0.11^a^	0.31 ± 0.06^f^
Control	5.49 ± 0.11^a^	1.08 ± 0.12^a^	0.26 ± 0.05^e^	1.48 ± 0.14^a^
L-carnitine	2.66 ± 0.09^d^	0.48 ± 0.06^c^	0.92 ± 0.05^c^	0.51 ± 0.04^d^
LF-CQPC04-L	3.63 ± 0.10^c^	0.74 ± 0.05^b^	0.56 ± 0.04^d^	0.70 ± 0.05^c^
LF-CQPC04-H	2.25 ± 0.12^e^	0.32 ± 0.03^d^	1.18 ± 0.07^b^	0.40 ± 0.03^e^
LB	4.02 ± 0.08^b^	0.77 ± 0.04^b^	0.51 ± 0.07^d^	0.93 ± 0.08^b^

**Group (liver tissue)**	**TG (mmol/gprot)**	**TC (mmol/gprot)**	**HDL-c (mmol/gprot)**	**LDL-c (mmol/gprot)**

Normal	4.41 ± 2.20^f^	72.83 ± 5.41^f^	65.21 ± 3.36^a^	0.42 ± 0.04^f^
Control	126.52 ± 8.71^a^	292.33 ± 16.12^a^	7.21 ± 1.08^f^	2.67 ± 0.21^a^
L-carnitine	33.25 ± 4.82^d^	162.34 ± 16.03^d^	36.25 ± 3.69^c^	0.92 ± 0.11^d^
LF-CQPC04-L	45.89 ± 6.33^c^	206.36 ± 19.32^c^	25.16 ± 4.08^d^	1.30 ± 0.12^c^
LF-CQPC04-H	19.78 ± 3.31^e^	126.71 ± 15.20^e^	48.32 ± 4.42^b^	0.68 ± 0.06^e^
LB	68.21 ± 7.82^b^	241.08 ± 21.35^b^	15.52 ± 2.81^e^	1.72 ± 0.09^b^

*Values presented are the mean ± standard deviation (*n* = 10/group). ^a–f^Mean values with different letters in the same column are significantly different (*P* < 0.05) according to Duncan’s multiple range test. LF-CQPC04-L: mice treated with a low concentration of *Lactobacillus fermentum* CQPC04 (1.0 × 10^8^ CFU/kg); LF-CQPC04-H: mice treated with a high concentration of *Lactobacillus fermentum* CQPC04 (1.0 × 10^9^ CFU/kg); LB: Mice treated with *Lactobacillus delbrueckii* subsp. *Bulgaricus* (1.0 × 10^9^ CFU/kg).*

### AST, ALT, and AKP Levels in the Serum and Liver Tissue of Mice

From Table 7, compared with the normal group, the content of ALT, AST, and AKP in the control group significantly increased (*P* < 0.05) after feeding a high-fat diet. Compared with the normal group, the content of ALT, AST, and AKP in the L-carnitine, LF-CQPC04-L, LF-CQPC04-H, and LB groups significantly decreased. The LF-CQPC04-H group showed the highest decrease.

**TABLE 7:** Serum and liver tissue levels of AST, ALT, and AKP in mice.

**Group (serum)**	**AST (U/L)**	**ALT (U/L)**	**AKP (U/L)**
Normal	8.30 ± 0.62^f^	5.03 ± 0.41^f^	112.51 ± 8.56^e^
Control	53.39 ± 2.12^a^	15.28 ± 0.43^a^	518.53 ± 23.65^a^
L-carnitine	20.68 ± 0.82^d^	8.39 ± 0.23^d^	356.20 ± 19.21^c^
LF-CQPC04-L	34.86 ± 1.04^c^	10.55 ± 0.63^c^	435.92 ± 27.98^b^
LF-CQPC04-H	14.28 ± 0.71^e^	7.62 ± 0.35^e^	229.61 ± 12.39^d^
LB	38.26 ± 1.14^b^	12.03 ± 0.52^b^	460.81 ± 33.02^b^

**Group (liver tissue)**	**AST (U/gprot)**	**ALT (U/gprot)**	**AKP (U/gprot)**

Normal	165.20 ± 12.41^f^	638.26 ± 20.36^f^	90.12 ± 5.69^f^
Control	2788.63 ± 121.05^a^	1271.86 ± 136.29^a^	282.09 ± 11.63^a^
L-carnitine	412.59 ± 28.05^d^	804.36 ± 18.16^d^	168.91 ± 13.68^d^
LF-CQPC04-L	791.29 ± 52.03^c^	870.22 ± 17.67^c^	191.22 ± 11.53^c^
LF-CQPC04-H	309.36 ± 23.56^e^	772.03 ± 20.37^e^	122.84 ± 16.70^e^
LB	1312.58 ± 119.23^b^	918.33 ± 16.08^b^	218.30 ± 14.58^b^

*Values presented are the mean ± standard deviation (*n* = 10/group). ^a–f^Mean values with different letters in the same column are significantly different (*P* < 0.05) according to Duncan’s multiple range test. LF-CQPC04-L: mice treated with a low concentration of *Lactobacillus fermentum* CQPC04 (1.0 × 10^8^ CFU/kg); LF-CQPC04-H: mice treated with a high concentration of *Lactobacillus fermentum* CQPC04 (1.0 × 10^9^ CFU/kg); LB: Mice treated with *Lactobacillus delbrueckii* subsp. *Bulgaricus* (1.0 × 10^9^ CFU/kg).*

### IL-6, IL-1β, TNF-α, IFN-γ, IL-4, and IL-10 Cytokine Levels in Mouse Serum

It can be seen from Table 8 that the content of four pro-inflammatory factors—IL-6, IL-1β, TNF-α, and IFN-γ—in the serum of the control group mice was significantly higher than those in the normal group (*P* < 0.05). L-carnitine, LF-CQPC04-L, LF-CQPC04-H, and LB groups were all significantly lower than the control group (*P* < 0.05). In addition, compared with the normal group, the levels of IL-4 and IL-10 in the control group were significantly lower, while the levels of the IL-4 and IL-10 in L-carnitine, LF-CQPC04-L, LF-CQPC04-H, and LB groups were higher than that in the normal group. Additionally, IL-6, IL-1β, TNF-α, and IFN-γ cytokine levels in the serum of the LF-CQPC04-H group mice were higher than those in the normal group but lower than those in other groups. Conversely, their IL-4 and IL-10 levels were lower than the normal group but higher than the other groups.

**TABLE 8:** Serum cytokine levels of IL-6, IL-1β, TNF-α, IFN-γ, IL-4, and IL-10 in mice.

**Group (serum)**	**IL-6 (pg/mL)**	**IL-1β (pg/mL)**	**TNF-α (pg/mL)**	**IFN-γ (pg/mL)**	**IL-4 (pg/mL)**	**IL-10 (pg/mL)**
Normal	45.39 ± 5.11^f^	14.32 ± 0.39^f^	17.08 ± 1.02^f^	5.60 ± 0.22^f^	70.26 ± 3.24^a^	135.62 ± 16.58^a^
Control	328.73 ± 19.54^a^	44.14 ± 0.67^a^	50.18 ± 2.62^a^	27.48 ± 0.67^a^	26.33 ± 2.64^f^	47.22 ± 3.15^f^
L-carnitine	118.36 ± 9.03^d^	26.01 ± 0.63^d^	34.19 ± 1.55^d^	11.28 ± 0.62^d^	55.17 ± 1.83^c^	84.53 ± 6.12^c^
LF-CQPC04-L	165.26 ± 12.17^c^	31.15 ± 0.48^c^	41.56 ± 1.71^c^	18.36 ± 0.72^c^	43.29 ± 1.56^d^	68.52 ± 5.22^d^
LF-CQPC04-H	98.36 ± 6.20^e^	19.30 ± 0.55^e^	25.18 ± 1.85^e^	8.19 ± 0.44^e^	63.12 ± 2.53^b^	108.92 ± 7.11^b^
LB	204.36 ± 15.23^b^	38.75 ± 0.48^b^	45.44 ± 1.28^b^	22.07 ± 0.60^b^	38.77 ± 2.03^e^	56.27 ± 3.66^e^

*Values presented are the mean ± standard deviation (*n* = 10/group). ^a–f^Mean values with different letters in the same column are significantly different (*P* < 0.05) according to Duncan’s multiple range test. LF-CQPC04-L: mice treated with a low concentration of *Lactobacillus fermentum* CQPC04 (1.0 × 10^8^ CFU/kg); LF-CQPC04-H: mice treated with a high concentration of *Lactobacillus fermentum* CQPC04 (1.0 × 10^9^ CFU/kg); LB: Mice treated with *Lactobacillus delbrueckii* subsp. *Bulgaricus* (1.0 × 10^9^ CFU/kg).*

### Pathological Observation of Liver and Epididymal Fat in Mice

**FIGURE 3:**
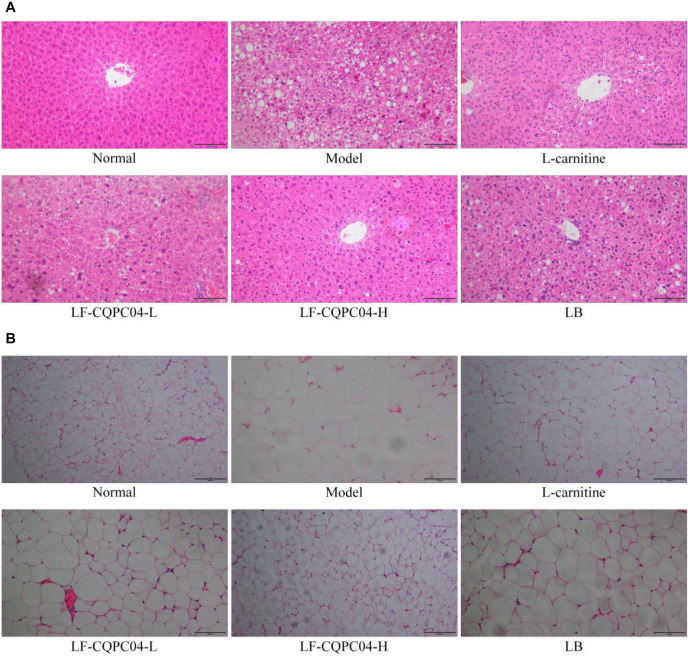
Pathological observation of H&E staining in mouse liver **(A)** and epididymal **(B)** tissues. LF-CQPC04-L: mice treated with a low concentration of *Lactobacillus fermentum* CQPC04 (1.0 × 10^8^ CFU/kg); LF-CQPC04-H: mice treated with a high concentration of *Lactobacillus fermentum* CQPC04 (1.0 × 10^9^ CFU/kg); LB: Mice treated with *Lactobacillus delbrueckii* subsp. *Bulgaricus* (1.0 × 10^9^ CFU/kg).

It can be seen from the slices (Figure 3A) that the liver cells of the control group mice were the most hypertrophic. They were sparsely secreted, arranged irregularly, and infiltrated by inflammatory cells. Conversely, the liver cells of the normal group mice were the most closely and evenly arranged. In the LF-CQPC04-H group, some liver cells show hypertrophy, and some local liver cells were sparsely distributed. The cell morphology was close to that in the normal group. Compared with the L-carnitine group, the liver cells in the LF-CQPC04-L group were sparsely distributed. The liver cells were larger, and the arrangement was more irregular. Conversely, the liver cells of LB group were even larger than those of the LF-CQPC04-L group; the distribution was sparser; and the arrangement was neat. Additionally, some cells showed inflammatory infiltration.

Slice observation (Figure 3B) shows that among the high-fat diet groups, epididymal adipocytes in the LF-CQPC04-H group were the smallest and most densely and regularly distributed. Epididymal adipocytes in the control group were the most hypertrophic and sparsely distributed. Compared with the LF-CQPC04-H group, epididymal adipocytes of the L-carnitine group were more hypertrophic, and the cells were more closely arranged and evenly distributed. Compared with the L-carnitine group, epididymal adipocytes in the LF-CQPC04-L group were more hypertrophic and sparsely distributed. Compared with the LF-CQPC04-L group, epididymal adipocytes in LB group were more hypertrophic and sparsely distributed.

### mRNA and Protein Expression of Cu/Zn-SOD, Mn-SOD, and CAT in Mice Liver Tissues

**FIGURE 4:**
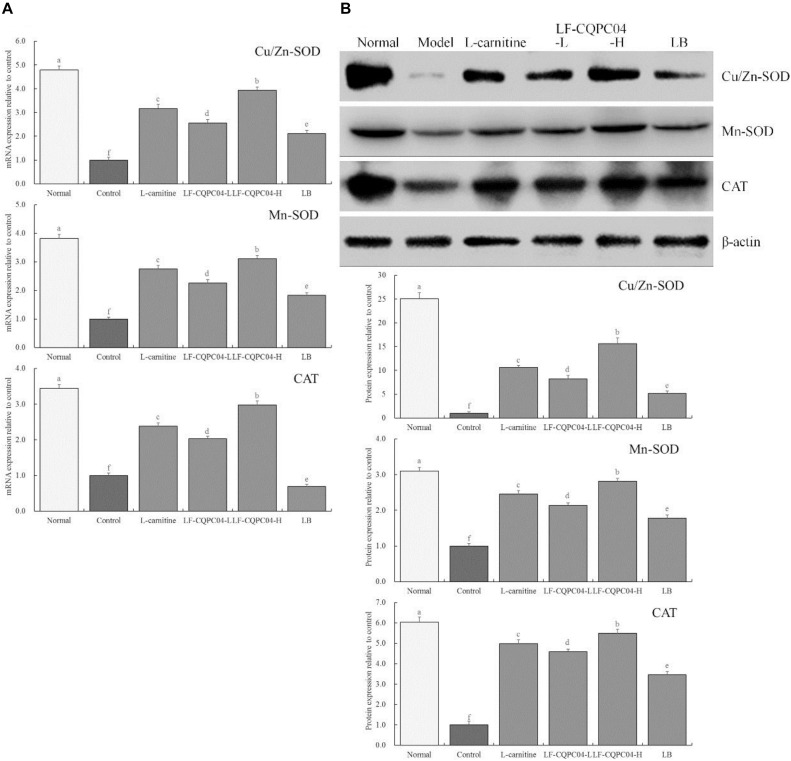
The mRNA **(A)** and protein **(B)** expression of Cu/Zn-SOD, Mn-SOD, and CAT in liver tissue of mice. ^a–f^Mean values with different letters in the same column are significantly different (*P* < 0.05) according to Duncan’s multiple range test. LF-CQPC04-L: mice treated with a low concentration of *Lactobacillus fermentum* CQPC04 (1.0 × 10^8^ CFU/kg); LF-CQPC04-H: mice treated with a high concentration of *Lactobacillus fermentum* CQPC04 (1.0 × 10^9^ CFU/kg); LB: Mice treated with *Lactobacillus delbrueckii* subsp. *Bulgaricus* (1.0 × 10^9^ CFU/kg).

Figure 4 shows that mRNA and protein expression of Cu/Zn-SOD, Mn-SOD, and CAT in the liver tissues of the control group mice were significantly (*P* < 0.05) lower than those of the other groups. Expression of Cu/Zn-SOD, Mn-SOD, and CAT in the liver tissues of mice in the LF-CQPC04-H group was only lower than that in the normal group, and the expression was significantly greater than that in the L-carnitine, LF-CQPC04-L, and LB groups.

### mRNA and Protein Expression of CYP7A1, PPAR-α, PPAR-γ, CPT1, LPL, C/EBP-α, and ABCA1 in Mice Liver Tissues

**FIGURE 5:**
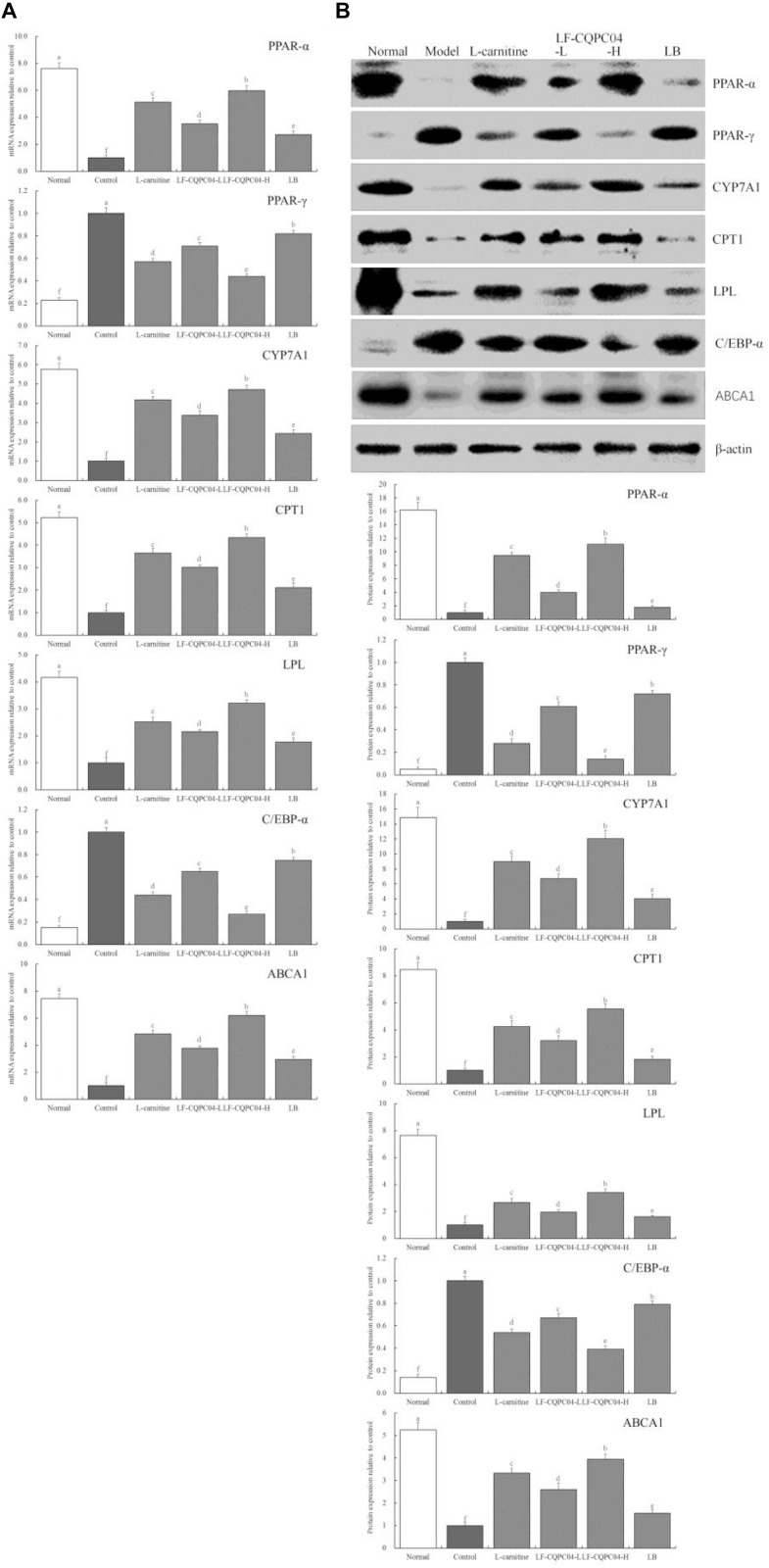
The mRNA **(A)** and protein **(B)** expression of PPAR-α, PPAR-γ, CYP7A1, CPT1, LPL, C/EBP-α, and ABCA1 in liver tissue of mice. ^a–f^Mean values with different letters in the same column are significantly different (*P* < 0.05) according to Duncan’s multiple range test. LF-CQPC04-L: mice treated with a low concentration of *Lactobacillus fermentum* CQPC04 (1.0 × 10^8^ CFU/kg); LF-CQPC04-H: mice treated with a high concentration of *Lactobacillus fermentum* CQPC04 (1.0 × 10^9^ CFU/kg); LB: Mice treated with *Lactobacillus delbrueckii* subsp. *Bulgaricus* (1.0 × 10^9^ CFU/kg).

Figure 5 shows that mRNA and protein expression intensity of CYP7A1, PPAR-α, CPT1, LPL, and ABCA1 in the liver tissue of the normal group mice was the greatest, and the expression intensity of PPAR-γ and C/EBP-α as the lowest. The mice from the control group presented the opposite trend. LF-CQPC04, LB, and L-carnitine significantly (*P* < 0.05) up-regulated the expression of CYP7A1, PPAR-α, CPT1, LPL, and ABCA1 in the liver tissue of obese mice (mice fed a high-fat diet) and down-regulated PPAR-γ and C/EBP-α expression, in which a high concentration of LF-CQPC04 (LF-CQPC04-H) showed the greatest ability to regulate these expressions.

### mRNA Expression of TNF-α and ZO-1 in Mice Colonic Tissues

**FIGURE 6:**
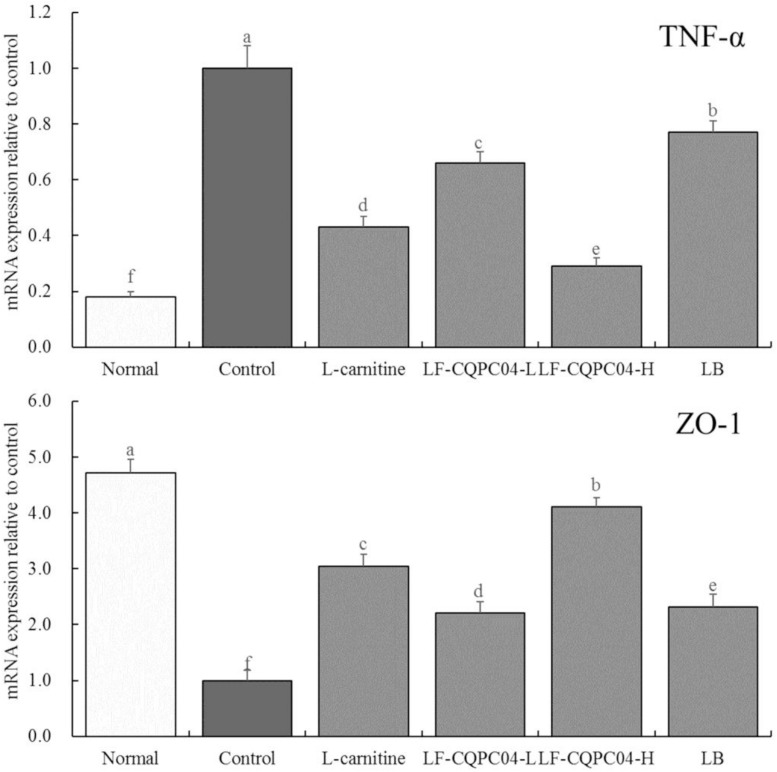
The mRNA expression of TNF-α and ZO-1 in colonic tissue of mice. ^a–f^Mean values with different letters in the same column are significantly different (*P* < 0.05) according to Duncan’s multiple range test. LF-CQPC04-L: mice treated with a low concentration of *Lactobacillus fermentum* CQPC04 (1.0 × 10^8^ CFU/kg); LF-CQPC04-H: mice treated with a high concentration of *Lactobacillus fermentum* CQPC04 (1.0 × 10^9^ CFU/kg); LB: Mice treated with *Lactobacillus delbrueckii* subsp. *Bulgaricus* (1.0 × 10^9^ CFU/kg).

Figure 6 shows that mRNA expression of TNF-α in the colonic tissue of the control group mice was the strongest, and the expression of ZO-1 was the weakest. LF-CQPC04, LB, and L-carnitine significantly (*P* < 0.05) down-regulated the expression of TNF-α in the colonic tissue of the control group mice and up-regulated ZO-1 expression. Moreover, the colon TNF-α and ZO-1 expressions of the LF-CQPC04-H group were the closest to those of the normal group.

### Microbial RNA Expression in Mouse Feces

**FIGURE 7:**
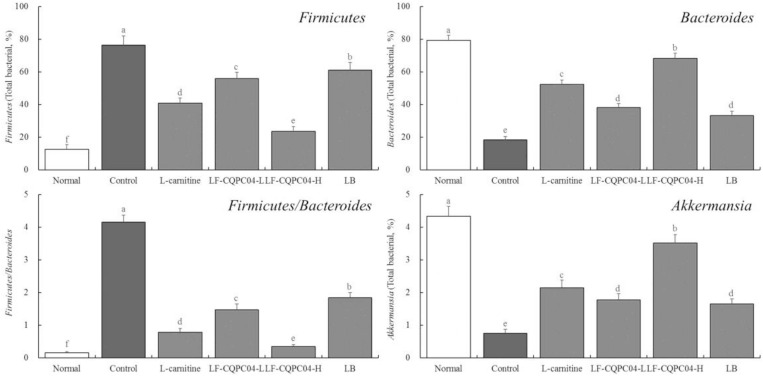
The mRNA expression in microorganisms in feces of mice. ^a–f^Mean values with different letters in the same column are significantly different (*P* < 0.05) according to Duncan’s multiple range test. LF-CQPC04-L: mice treated with a low concentration of *Lactobacillus fermentum* CQPC04 (1.0 × 10^8^ CFU/kg); LF-CQPC04-H: mice treated with a high concentration of *Lactobacillus fermentum* CQPC04 (1.0 × 10^9^ CFU/kg); LB: Mice treated with *Lactobacillus delbrueckii* subsp. *Bulgaricus* (1.0 × 10^9^ CFU/kg).

In the feces of the control group mice, the expression of *Firmicutes* was the strongest; the expression of *Bacteroides* and *Akkermansia* was the weakest; and the ratio of *Firmicutes/Bacteroides* was the highest (*p* < 0.05). Compared with the control group mice, the LF- CQPC04-, LB-, and L-carnitine-treated mice showed lower *Firmicutes* levels and *Firmicutes/Bacteroides* ratios, but higher *Bacteroides* and *Akkermansia* levels (Figure 7). Interestingly, the microbial community in the feces of the LF-CQPC04-H group resembled that of healthy mice.

## Discussion

Obesity is a common disease. It is a manifestation of excessive body fat and is closely related to lipid metabolism disorders. Lipid metabolism disorders can be manifested as follows: excessive intake and a high-energy diet increase fat synthesis; reduction of brown fat content leads to the reduction of energy consumption; and abnormal regulation of lipid-lowering hormones causes the increase of fat syntheses and the reduction of fat degradation (Stern, 1984). Obesity primarily manifests as an excess in body fat content, increase in volume of fat cells, body fat distribution disorder, and local fat deposition. Furthermore, most obese patients have severe lipid metabolism disorders, which often coexist with diabetes, coronary heart diseases, and hypertension. It is called the “metabolic syndrome” and has become an important cause of disease (Cai, 2013). Obesity affects lipid metabolism in many ways. Food structure, food intake and lifestyle are factors that affect lipid metabolism. Genetic factors that affect lipid metabolism include certain hormones, cytokines, and enzymes (Wilding, 1997).

The direct manifestation of obesity caused by lipid metabolism disorder is abnormal weight. Animal experiments showed abnormal visceral mass, resulting in abnormal changes in visceral index (Zhu et al., 2019). Additionally, visceral tissue lesions caused by abnormal increases in lipid content can be intuitively judged from pathological observation (Zhao et al., 2019). In this study, a high-fat diet caused abnormal weight gain in mice. Their visceral index was also significantly different from the normal state. Moreover, pathological observation showed liver cell lesions. LF-CQPC04 can maintain body weight and visceral index and reduce abnormalities caused by a high-fat diet better than LB. In addition, it protects liver cells and prevents liver tissue lesions caused by increased lipid content.

In obesity caused by elevated lipid levels, increased fat mobilization increases free fatty acids, TC, and TG in the blood. It also reduces VLDL and LDL-c clearance and HDL-c to a level below the normal value (Ohno et al., 1983; Carrier et al., 2014). The liver plays an important role in lipid digestion, absorption, decomposition, synthesis, and transport. The liver can secrete bile, and the bile acid salt is the conversion product of cholesterol in the liver. It can emulsify lipids, as well as promote the digestion and absorption of lipids (Park et al., 2018). Alanine aminotransferase (ALT), aspartate aminotransferase (AST), and alkaline phosphatase (AKP) are important indicators in the diagnosis of liver function. These indicators will be obviously abnormal in the case of liver damage. Those with abnormal liver function cannot metabolize fat normally, which leads to obesity (Zhu et al., 2019). This study again proves that LF-CQPC04 can regulate lipid metabolism and ease liver dysfunction caused by abnormal lipid levels.

IL-6 and TNF-α can regulate the synthesis and secretion of hepatocyte apolipoprotein by affecting the expression of 3-hydroxy-3-methylglutaryl-CoA reductase (HMGR). In addition, these cytokines can induce lipid peroxidation and changes in lipid structure and function of cells (Pickens et al., 2017). IL-1β can affect the activity of paraoxonase 1 and reduce the expression of paraoxonase 1 in hepatocytes cultured *in vitro*. Changes in paraoxonase 1 during the acute reaction period will cause high-density lipoproteins to lose their anti-inflammatory efficacy (Khalkhal et al., 2012). IL-10 can regulate lipid levels at the genetic level. IL-10 protein molecules directly affect the secretion of high-density lipoprotein cholesterol. The genotypes of different protein expression levels affect HDL cholesterol levels by controlling IL-10 levels. IL-10 gene polymorphism affects the inflammatory response process and secretion of other inflammatory factors through different protein expression levels, which indirectly affects HDL-c levels (Jung et al., 2008). Excessive intake of saturated fatty acids is the main cause for the increase of blood cholesterol, triacylglycerol, and low-density lipoprotein cholesterol. It is also a secondary cause for arterial luminal stenosis, which leads to the formation of atherosclerosis and increases the risk of coronary heart diseases (Kim and Wierzbicki, 2020). Palmitic acid is a typical saturated fatty acid. The content of fatty acids in monocytes activated by IFN-γ significantly increased, especially palmitic acid. This leads to lipid metabolism dysfunction, which can be harmful to the human body (Zhang C. et al., 2018). There is a unique type 1 fat-sensitive natural killer cell (NKT) in adipose tissue, whose concentration decreases with the degree of obesity. NKT cells in the adipose tissue play a key role in regulating TH1/TH2 response of adipose tissue, macrophage polarization, and glucose homeostasis. IL-4 mediated TH2 immune response participates in the regulation of fat by NKT cells, promoting the growth of adipose tissue and affecting lipid metabolism (Szczepankiewicz et al., 2018). In this study, LF-CQPC04 had a significant effect on these cytokines, thereby regulating lipid metabolism and promoting mice to return to normal weight.

A high-energy diet will not only cause a large amount of fat accumulation, resulting in an imbalance in lipid metabolism, but it will also affect the body’s redox balance (Luo et al., 2020). A high-energy diet induces hyperglycemia through lipid metabolism and sugar metabolism and, subsequently, releases a large amount of free radicals, triggering oxidative stress (Park et al., 2020). Abnormal lipid metabolism caused by a high-energy diet will change the normal secretion of endogenous hormones and prevent free radicals generated by the body from being quickly and effectively eliminated, exacerbating oxidative stress (Rana et al., 2019). By improving the activity of SOD enzyme in the liver, lipid peroxide in the liver can be reduced, thereby reducing the surplus of energy in the body, avoiding fat accumulation, and promoting normal lipid metabolism, and sugar metabolism (Shin et al., 2019; Wu et al., 2020; Yi et al., 2020; Wu et al., 2017). Meanwhile, controlling the balance of free radicals in the body and maintaining redox balance can avoid cell damage and the formation of inflammatory cytokines IL-6, IL-1β, TNF-α, IFN-γ, IL-4, and IL-10, which regulate lipid metabolism (Hu et al., 2019). SOD can be divided into three categories, namely, Cu/Zn-SOD, Mn-SOD, and Fe-SOD, based on the metal prosthetic groups. There are Cu/Zn-SOD and Mn-SOD but no Fe-SOD in vertebrates (Donnelly et al., 1989). SOD (Cu/Zn-SOD and Mn-SOD) and CAT are both important antioxidant enzymes in the body and play a role in scavenging free radicals (Zhou et al., 2019). In this study, LF-CQPC04 also showed significant effects on regulating Cu/Zn-SOD, Mn-SOD, and CAT. Therefore, it may play a role in maintaining normal lipid levels by regulating oxidative stress.

CYP7A1 is a rate-limiting enzyme that catalyzes the breakdown of cholesterol into bile acids in the liver. It is regulated by a variety of factors to maintain balance in the cholesterol metabolism. Regulating the expression of CYP7A1 can significantly affect the process of lipid metabolism (Wang et al., 2020). PPAR-α belongs to the peroxisome proliferator-activated receptor family. It is widely expressed in various organs and tissues, mainly controlling fatty acid oxidation and energy decoupling in adipose tissue and muscle. It inhibits macrophage-induced inflammation and participates in the decomposition of fatty acids. It plays an important role in restoring normal lipid levels (Naseri et al., 2020). Some humoral factors in obese patients, including PPAR-γ, can cause abnormal regulation of adipocyte differentiation, proliferation, and metabolism. PPAR-γ is a nuclear receptor protein that induces adipocyte differentiation by stimulating the expression of several genes involved in preadipocyte proliferation. Activated PPAR-γ terminates the cell differentiation cycle and stimulates the expression of adipocyte-specific genes, resulting in increased cellular energy uptake (Shi et al., 2020). CPT1 is a rate-limiting enzyme in the beta oxidation process of fatty acids. It is located in the outer mitochondrial membrane. It catalyzes the transfer of long-chain fatty acids from acyl-CoA to carnitine, which then enters the mitochondria from the cytoplasm. Carnitine participates in β-oxidation catalyzed by CPT2 located on the inner mitochondrial membrane.

In addition, CPT1 can determine the content of acyl-CoA in the cytoplasm, thereby regulating lipid levels (Lei et al., 2017). When insulin receptors on mast cells are insensitive to insulin or the number of receptors is reduced, both factors can reduce the activity of insulin-dependent lipoprotein lipase (LPL), resulting in reduced lipolysis. This is the root cause of lipid metabolism disorders in obese patients. LPL is a TG hydrolase and plays a key role in the catabolism of TG-rich VLDL and chylomicrons. Decreased LPL activity will reduce the decomposition of VLDL, which leads to the elimination of excess lipids. LDL in obese individuals can easily form small and dense particles, and it can be quickly oxidized, which is not conducive to clearing. It will also reduce cholesterol content (Mesilati-Stahy and Argov-Argaman, 2018). C/EBP-α is a transcription factor that regulates the differentiation of adipocytes. It can interact with PPAR-γ, and its expression is positively correlated with the expression of PPAR-γ (Muni Swamy et al., 2020). There is a PPARγ-LXRα-ABCA1 pathway in the expression of ABCA1. PPARγ activates LXRα expression and then acts on target genes to increase ABCA1 expression, which mediates reverse cholesterol transport in cells and reduces lipid accumulation in the body (Zhao et al., 2018). The experimental results show that LF-CQPC04 can effectively regulate the expression of CYP7A1, PPAR-α, PPAR-γ, CPT1, LPL, C/EBP-α, and ABCA1, thereby regulating lipid metabolism and easing abnormal lipid levels in mice caused by a high-fat diet.

Obesity caused by a high-fat diet will lead to intestinal barrier damage, increased permeability and increased inflammation, especially in the colon (Yu et al., 2019). TNF-α is an important regulatory factor in the body, which can promote inflammatory response and immune regulation. It can further aggravate inflammation in the colon by stimulating the synthesis and release of interleukin-1 and interleukin-8 in monocytes and macrophages (Wang et al., 2016). ZO-1 is one of the important components of tight junctions. Many transmembrane proteins and cytoplasm need to be connected by tight junction proteins, in order to regulate cell permeability. Hypoxia and inflammatory injury may lead to abnormal distribution, decreased expression, and even dissolution of ZO-1 protein, resulting in widening of cell space, damage of tight junction structure, and increased permeability of the endothelial layer, which eventually leads to aggravation of inflammation in the colon (Peng et al., 2018). In this study, LF-CQPC04 can regulate these TNF-α and ZO-1 expressions in colon tissue to a normal state, which shows that LF-CQPC04 can restore normal intestinal function by reducing lipid levels.

Studies have shown that a high-fat can lead to intestinal flora imbalance, and the reduction of beneficial bacteria in the intestine will further instigate lipid metabolism disorder, aggravate lipid increase in the body, and trigger inflammation (Zhang Y. et al., 2018). The results showed that LF-CQPC04 reduced the number of harmful bacteria (e.g., *Firmicutes*) and increased the number of beneficial bacteria (e.g., *Bacteroides* and *Akkermansia*) (Shen et al., 2020). This indicates that LF-CQPC04 could regulate the growth of probiotics, inhibit the growth of harmful bacteria, and also regulate the ratio of *Firmicutes/Bacteroides* to increase the diversity of intestinal flora communities and restore the health of intestinal flora. Thus, LF-CQPC04 can inhibit the increase of lipids in the body and promote the balance of intestinal microflora.

## Conclusion

In this study, we observe the regulatory effects of a newly discovered strain of LF-CQPC04 on lipid reduction by establishing a mouse control of abnormal lipid levels. The results show that LF-CQPC04 can effectively alleviate elevated lipids disorder caused by a high-fat diet in mice and inhibit diseases, including liver injury. The effects are better than that of L-carnitine, which is commonly used in weight loss and lipid level regulation. Moreover, the effects of LF-CQPC04 are superior to the commonly used commercial strain LB. Further research also shows that LF-CQPC04 can regulate lipid levels and protect the body by restoring liver function. LF-CQPC04 can also reduce inflammation and oxidative stress, as well as regulate the PPAR-α signaling pathways. LF-CQPC04 also can reduce colitis caused by a high-fat diet and regulate intestinal flora. LF-CQPC04 is a high-quality microbial resource with significant probiotic value. LF-CQPC04 has shown potential for further application as a probiotic or a food. However, this study only carried out *in vivo* animal studies. Moreover, it lacks corresponding cell experiments to verify the molecular mechanism and human experiments to verify the effect. In future studies, these experiments will be carried out to better prove the effect of LF-CQPC04.

## Data Availability Statement

The raw data supporting the conclusions of this article will be made available by the authors, without undue reservation.

## Ethics Statement

The animal study was reviewed and approved by Ethics Committee of Chongqing Collaborative Innovation Center for Functional Food (201905020B).

## Author Contributions

RY and FT performed the majority of the experiments and wrote the manuscript. XRZ, JM, LL, and XD contributed to the data analysis. ZY and XZ designed and supervised the study, and checked the final manuscript. All authors contributed to the article and approved the submitted version.

## Conflict of Interest

The authors declare that the research was conducted in the absence of any commercial or financial relationships that could be construed as a potential conflict of interest.
